# Constitutive activation of BMP signalling abrogates experimental metastasis of OVCA429 cells via reduced cell adhesion

**DOI:** 10.1186/1757-2215-3-5

**Published:** 2010-02-26

**Authors:** Trevor G Shepherd, Michelle L Mujoomdar, Mark W Nachtigal

**Affiliations:** 1 Department of Pharmacology, Dalhousie University, Sir Charles Tupper Medical Building, Halifax, NS, Canada; 2 Cancer Research Laboratory Program, London Health Sciences Centre, 790 Commissioners Rd E, London, ON, Canada; 3 Department of Obstetrics & Gynaecology, The University of Western Ontario, London, ON, Canada; 4 Department of Oncology, The University of Western Ontario, London, ON, Canada; 5 Department of Anatomy & Cell Biology, The University of Western Ontario, London, ON, Canada

## Abstract

**Background:**

Activation of bone morphogenetic protein (BMP)4 signalling in human ovarian cancer cells induces a number of phenotypic changes *in vitro*, including altered cell morphology, adhesion, motility and invasion, relative to normal human ovarian surface epithelial cells. From these *in vitro *analyses, we had hypothesized that active BMP signalling promotes the metastatic potential of ovarian cancer.

**Methods:**

To test this directly, we engineered OVCA429 human ovarian cancer cells possessing doxycycline-inducible expression of a constitutively-active mutant BMP receptor, ALK3^QD^, and administered these cells to immunocompromised mice. Further characterization was performed *in vitro *to address the role of activated BMP signalling on the EOC phenotype, with particular emphasis on epithelial-mesenchymal transition (EMT) and cell adhesion.

**Results:**

Unexpectedly, doxycycline-induced ALK3^QD ^expression in OVCA429 cells reduced tumour implantation on peritoneal surfaces and ascites formation when xenografted into immunocompromised mice by intraperitoneal injection. To determine the potential mechanisms controlling this *in vivo *observation, we followed with several cell culture experiments. Doxycycline-induced ALK3^QD ^expression enhanced the refractile, spindle-shaped morphology of cultured OVCA429 cells eliciting an EMT-like response. Using *in vitro *wound healing assays, we observed that ALK3^QD^-expressing cells migrated with long, cytoplasmic projections extending into the wound space. The phenotypic alterations of ALK3^QD^-expressing cells correlated with changes in specific gene expression patterns of EMT, including increased Snail and Slug and reduced E-cadherin mRNA expression. In addition, ALK3^QD ^signalling reduced β1- and β3-integrin expression, critical molecules involved in ovarian cancer cell adhesion. The combination of reduced E-cadherin and β-integrin expression correlates directly with the reduced EOC cell cohesion in spheroids and reduced cell adhesion to the extracellular matrix substrates fibronectin and vitronectin that was observed.

**Conclusions:**

We propose that the key steps of ovarian cancer metastasis, specifically cell cohesion of multicellular aggregates in ascites and cell adhesion for reattachment to secondary sites, may be inhibited by overactive BMP signalling, thereby decreasing the ultimate malignant potential of ovarian cancer in this model system.

## Background

Ovarian cancer is the most lethal of the gynaecologic malignancies in the Western world. The majority of ovarian cancers are detected as late-stage disease and involve the dissemination of tumour cells throughout the peritoneal cavity and the production of ascites; these clinical assessments are correlated with a very poor prognosis (only 5-40% five-year survival) for patients[1]. Successful early detection and more effective management of late-stage disease are crucial to improving the survival and quality-of-life of ovarian cancer patients. Understanding the underlying molecular mechanisms of ovarian cancer pathogenesis is key to achieving this goal.

Previous work from our laboratory demonstrated that normal human ovarian surface epithelial (OSE) cells and human epithelial ovarian cancer (EOC) cells possess an intact autocrine bone morphogenetic protein-4 (BMP4) signalling pathway[2]. BMPs comprise approximately 20 unique members of the transforming growth factor-beta (TGFβ) superfamily of cytokines[3]. BMPs act as extracellular dimeric ligands by binding to the type I (ALK2, ALK3, and ALK6) and type II (BMPR2) receptors[4]. BMP signalling is mediated via a heterotetrameric receptor complex composed of a type I receptor that is phosphorylated at the intracellular GS domain by type II receptor serine/threonine kinase activity, leading to the association of receptor-activated Smad (R-Smad) proteins. Upon phosphorylation, the BMP-specific R-Smads (Smads 1, 5 and 8) dimerize and associate with the common-mediator Smad, Smad4. This activated Smad complex translocates to the nucleus and regulates the transcription of target genes, typically via its interaction with several other transcription factors and co-activator and co-repressor proteins[5]. Recent work also shows that Smad independent signalling can be initiated by the activated receptor complex[4,6,7].

The multifaceted and complicated roles of TGFβ signalling in the pathogenesis of many human cancers is well established[8,9], yet our understanding of the contribution of BMP signalling to cancer biology is limited. In many instances, activation of BMP signalling inhibits cell growth and induces apoptosis in different cancer cell types[10-16]. The identification of inactivating germline mutations in the human *BMPR1A *gene (encoding the ALK3 receptor) in juvenile polyposis patients indicates a putative tumour suppressor function of active BMP signalling in colon cancer[17,18]. However, other studies have found that BMP signalling may be implicated in increasing metastatic potential[19-21] and tumour angiogenesis[22]. While there has been some advancement in our understanding of the functional implications of BMP signalling in various cancers, the contribution of BMP signalling to ovarian cancer pathogenesis requires further clarification.

Treatment of primary human OSE and EOC cells with exogenous BMP4 ligand results in a cell spreading phenotype and increased cell adhesion[2,23]. Furthermore, BMP4 induces an epithelial-mesenchymal transition (EMT) morphologic response in primary EOC cells isolated from patient ascites by increasing Snail and Slug expression and a subsequent decrease in E-cadherin [23]. BMP4 directly upregulates *ID1 *and *ID3 proto*-oncogene expression in EOC cells compared with normal OSE[2], and BMP4 signalling increases EOC cell motility whereas normal OSE cell motility is unaffected[23]. We sought to develop a model system to elucidate the function of active BMP signalling in ovarian cancer metastasis. We chose to use the human ovarian cancer cell line OVCA429 which is capable of producing ascites and peritoneal implants mimicking the spread of ovarian cancer observed in patients [24]. To this end, we employed a doxycycline (Dox)-inducible expression system in OVCA429 cells to ectopically express a constitutively-active mutant BMP type I receptor (ALK3^QD^). Herein, we report that constitutively-active ALK3 receptor signalling decreases the intraperitoneal dissemination of OVCA429 cells in nude mouse xenografts. We provide further evidence that this may occur via downregulation of E-cadherin and β1-/β3-integrin expression thereby reducing cell-cell cohesion and cell-substratum adhesion. The application of this model system to an *in vivo *context provides insight into how cellular responses affected by constitutive BMP signalling directly impacts ovarian cancer metastasis.

## Methods

### Cell culture

CaOV3 and SkOV3 human ovarian cancer cells were grown in Dulbecco's modified Eagle medium (Invitrogen) containing 10% heat-inactivated fetal bovine serum (FBS; Hyclone). OVCA429 human ovarian cancer cells (gift of Dr. B. Vanderhyden, Ottawa Regional Cancer Centre) were grown in Minimal essential medium Eagle-alpha (Invitrogen) containing 10% FBS and supplemented with 0.1 mM non-essential amino acids (Invitrogen). Primary cultures of human OSE and EOC cells were isolated and maintained as previously described[25]. Treatment of OVCA429 cells with recombinant human BMP4 (R&D Systems) was performed as described previously[2]. Institutional approval for research with human materials was received prior to the initiation of these studies (QEII Health Sciences Centre, Research Ethics Committee, #QE-RS-99-016; IWK/Grace Hospital Research Ethics).

OVCA429 Tet-On cells (429T cells) were generated by transfecting OVCA429 cells with pTet-On vector (Clontech) using GeneJuice reagent (Novagen), followed by selection with 1 mg/mL Geneticin™ (Invitrogen). Of the 27 separate clones generated, 5 clones displayed detectable doxycycline-inducible activity, as assessed by transient transfection of the pTRE2-*luc *vector and followed by luciferase assays performed as previously described[2]. Doxycycline hyclate (Dox; Sigma) was used at a concentration of 2 μg/mL in all experiments, unless otherwise indicated. Dox-inducible ALK3^QD^-expressing cells (429T-ALK3^QD^) were generated by transfecting 429T cells with pTRE2-ALK3^QD^-HA (original pCMV5-Alk3QDHA plasmid was a gift from Dr. L. Attisano, U. of Toronto), and selection with 250 μg/mL Hygromycin B (Invitrogen). Five different 429T-ALK3^QD ^cell lines possessing Dox-inducible ALK3^QD ^expression were identified by Western blot analysis, and two independent clones (clones 429T-A44 and -54) were chosen for further examination. Subsequent growth of 429T and 429T-ALK3^QD ^cell lines was maintained using 100 μg/mL Geneticin™ and 100 μg/mL Hygromycin B.

### Northern analysis

Northern analysis was performed as previously described[2], using radiolabelled probes synthesized from human cDNA fragments of *ALK3 *(nucleotides 145-594), *ALK6 *(nucleotides 907-1256), *BMPR2 *(nucleotides 2561-3010), *GAPDH *(nucleotides 270-620), *ID1 *(nucleotides 189-549), and *ID3 *(nucleotides 356-750) as templates.

### Western analysis

Protein isolation and subsequent Western analyses were performed as previously described[2]. ALK3^QD ^protein expression was detected using anti-HA-Peroxidase 3F10 monoclonal antibody (1:1000 dilution; Roche). The anti-actin polyclonal antibody (1:1000 dilution; Sigma) followed by incubation with horseradish peroxidase-conjugated sheep anti-rabbit IgG secondary antibody (1:5000 dilution; Chemicon) was used as a control for protein loading.

### Tumour cell xenografting

All studies conformed to the approved animal utilization protocol and Canadian Council for Animal Care guidelines. Eight- to 10-week-old female CD-1 *nu*/*nu *athymic nude mice (Charles River) were maintained in a sterile barrier animal facility. After approximately one week from receipt, 30 mice were started on a diet of gamma-irradiated rodent chow containing Dox at a concentration of 1000 ppm (Research Diets) for 2 d, and another group of 30 mice remained on the in-house rodent chow diet. Mice were supplied with food and water *ad libitum*. Each mouse was injected i.p. with a suspension of 5 × 10^5 ^cells (either 429T-ALK3^QD ^or 429T control) in a volume of 0.2 mL sterile 1 × PBS, resulting in four groups of fifteen mice (429T-ALK3^QD ^or 429T controls, with or without Dox treatment). Mice were monitored for 90 d post-injection for signs of ascites formation or visible morbidity. At the experimental endpoint (90 d), mice were sacrificed and dissection was performed to assess and quantify ascites production and tumour formation.

### Histology

Tissues harvested for histological analysis were fixed immediately in 4% paraformaldehyde/1 × PBS, paraffin-embedded and sectioned at 5 μm then stained with haematoxylin and eosin (tissue processing performed by Molecular Pathology, Robarts Research Institute, UWO). Microscopic images of stained tissue sections were captured using an Olympus IX70 inverted microscope and Image Pro 6.2 software, and subsequently adjusted for brightness/contrast and colour balance using Adobe Photoshop 7.0 software.

### RT-PCR

Confirmation of *ALK3*^*QD *^transgene expression in tumours that formed in nude mice was performed by RT-PCR analysis of total RNA isolated from homogenized tissue using the Total RNA isolation kit (Sigma) as per manufacturer's instructions. Reverse transcription was performed with 2 μg of RNA reverse transcribed into cDNA using oligo-dT decamers as primers and Superscript II reverse transcriptase (Invitrogen) as per manufacturer's instructions. Subsequent PCR was carried out using oligonucleotides that specifically amplify the 3' end of the ALK3^QD^-HA cDNA construct. Human *GAPDH *mRNA expression served as a control for tumour xenograft material in each sample.

For quantitative RT-PCR analysis, total RNA was isolated from cells treated with 2 μg/mL Dox for 2 days, or left untreated, and 2 μg of RNA was subsequently reverse transcribed into cDNA using oligo-dT decamers as primers and Superscript II reverse transcriptase (Invitrogen) as per manufacturer's instructions. PCR was performed using the Brilliant SYBR green QPCR Master Mix (Stratagene), and real-time measurement of the PCR reactions was recorded using the Mx3000P Real-time PCR System (Stratagene), and quantified using the 2^-ΔΔCt ^method[26]; *GAPDH *expression was used for normalization, and the fold change in mRNA expression was calculated *versus *untreated cell samples.

All human gene-specific primer sequences used in RT-PCR are available upon request.

### Scratch wound assays

Cells were seeded at 2 × 10^5 ^cells in 6-well dishes and 24 h later were treated with 2 μg/mL Dox, or left untreated. Cells were grown for 2-3 d until confluent monolayers were achieved, then a wound was created by scratching the wells with a sterile plastic pipette tip (~1 mm space). After several gentle washes with 1 × PBS, media was replaced (with or without Dox) and cells were monitored at multiple timepoints and photographed at 24 h. Photographic images were captured using a Nikon Coolpix digital camera and Nikon inverted phase contrast microscope at 100 × magnification.

### Adhesion assays

For assessment of cell detachment, cells were seeded at 5 × 10^5 ^cells in 6-well dishes, and 24 h later were treated with 2 μg/mL Dox, or left untreated. Detached cells were quantified as previously described[2].

In adhesion experiments, cell lines were treated with 2 μg/mL Dox, or left untreated, and grown for 2-3 d until confluent monolayers were achieved. Adhesion assays were performed as previously described[27]. Cells were plated at a density of 1 × 10^5 ^cells/well into 24-well dishes previously coated with 500 ng/cm^2 ^fibronectin or 200 ng/cm^2 ^vitronectin (Sigma), and blocked with 1% BSA.

### Spheroid formation

429T and 429T-ALK3^QD ^cells were pre-treated with 2 μg/mL Dox for 2 days, or left untreated, while grown in monolayer culture. One hundred thousand cells/well were then seeded in quadruplicate into a 24-well Ultra-Low Attachment cluster plate (Corning) and the same culture conditions (*i.e*. +/- Dox) were maintained during spheroid formation. Images were captured at the 2-day timepoint using an Olympus IX70 inverted microscope at 100 × magnification and Image Pro 6.2 software.

### Statistical analyses

Statistical analyses were performed using the nonparametric Mann-Whitney test with 95% confidence intervals (GraphPad Prism 4). Values of significance are indicated in the Legends to Figures.

## Results

### BMP receptor expression in OSE and EOC cells

**Figure 1:**
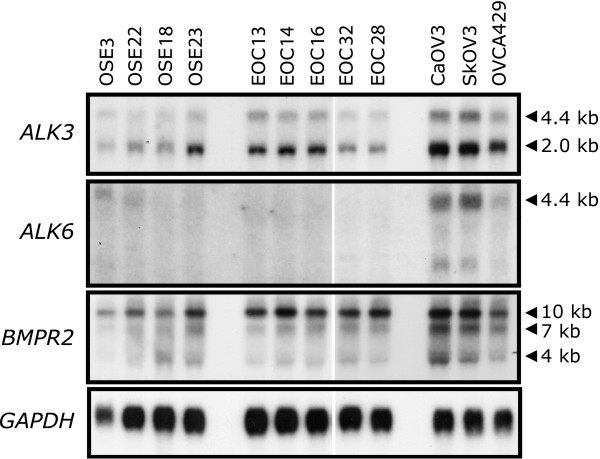
**BMP receptor expression in normal human OSE and human ovarian cancer cells**. Northern blot analysis was performed on total RNA isolated from early-passage primary cultures of normal human OSE cells (OSE3, OSE18, OSE22, and OSE23), primary human EOC cells (EOC13, EOC14, EOC16, EOC28, and EOC32), and three established human ovarian cancer cell lines (CaOV3, SkOV3, and OVCA429) using probes specific for *ALK3*, *ALK6*, and *BMPR2 *mRNA. *ALK3 *and *BMPR2 *mRNA expression was readily detectable (24 h exposure) in all samples analysed, whereas *ALK6 *mRNA expression was observed only in ovarian cancer cell lines after an extended period of time (8 d exposure). Expression of all three BMP receptors was elevated in the three cell lines *versus *the primary cultures; however, there was no mean difference in receptor expression between primary cultures of OSE and EOC cells.

Previous work from our laboratory demonstrated that primary cultures of normal human OSE cells and EOC cells possess an intact BMP4 signalling pathway, but more importantly, that primary EOC cells exhibit striking changes in morphology, adhesion, motility and invasion in response to BMP4 stimulation[2,23]. Additionally, EOC cells exhibit heightened responses in gene expression following treatment with exogenous BMP4 in comparison to normal OSE cells; for example, *ID1 *gene expression is increased ~10- to 15-fold in EOC cells, compared to 2- to 3-fold in normal OSE[2,23]. To further our understanding of the potential differential responses to BMP4 signalling in these cell types, we quantified BMP receptor expression levels in normal OSE and EOC cells. BMP4 signalling is mediated primarily by the ALK3, ALK6, and BMPR2 receptors. In Northern blot analyses using RNA isolated from actively growing primary cultures of OSE and EOC cells, and the EOC cell lines CaOV3, SkOV3, and OVCA429, we readily detected expression of the type I BMP receptor *ALK3 *and the type II receptor *BMPR2 *mRNA (Fig. 1). After normalization to *GAPDH *expression, no significant differences in the mRNA level of *ALK3 *or *BMPR2 *were detectable when normal OSE is compared with primary EOC samples. Expression of *ALK6 *mRNA was largely undetectable in all primary cell samples. Additionally, we have determined that BMPR2 protein expression as well as the BMP-specific R-Smads, Smad1 and Smad5, remain unchanged between primary OSE and EOC cells (T Shepherd & M Nachtigal, unpublished observations). By comparison, immortalized EOC cell lines expressed higher levels of *ALK3, ALK6*, and *BMPR2 *than the primary cell samples. These data are consistent with quantitative RT-PCR results (data not shown) suggesting that ALK3 and BMPR2 are likely the chief receptors used by BMP4 to confer activated signalling in primary cultures of human OSE and EOC cells.

### Constitutive ALK3 receptor signalling reduces metastatic potential *in vivo*

**Figure 2:**
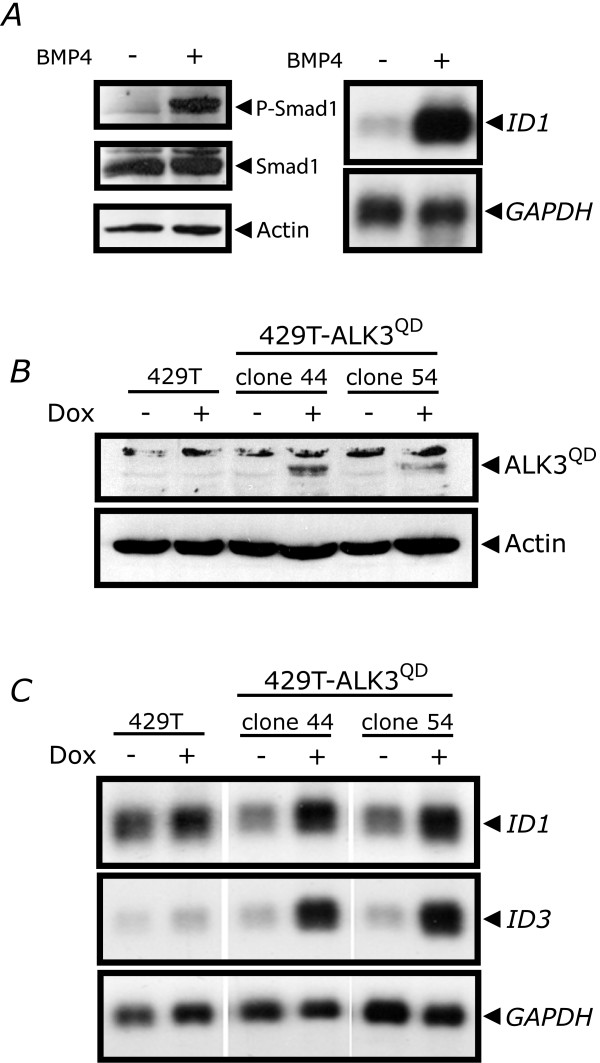
**Generation of human ovarian cancer OVCA429 cells with Dox-inducible expression of the constitutively-active mutant ALK3 receptor (ALK3^QD^)**. (A) Phosphorylation of Smad1 (P-Smad1) and expression of *ID1 *mRNA are induced in serum-starved OVCA429 cells treated with 10 ng/mL BMP4 for 30 min and 90 min, respectively. Total Smad1 and actin were used as protein loading controls, and *GAPDH *for RNA loading control. (B) Expression of HA-tagged constitutively-active ALK3 receptor (ALK3^QD^) was observed by Western analysis in two independent 429T-ALK3^QD ^stable cell clones (A44 and A54) after 24 h treatment with Dox, as compared with control cells (T7Hyg4). Actin was used as a control for protein loading. (C) Activated BMP signalling was confirmed by Northern analysis of *ID1 *and *ID3 *mRNA expression from 429T-A44 and 429T-A54 cells, and 429T control cells, treated with Dox for 24 h, or left untreated. *GAPDH *served as a control for RNA loading.

Our goal was to examine the consequence of BMP signalling activity on the metastatic potential of ovarian cancer cells. We engineered OVCA429 cells that possess Dox-inducible expression of the mutated BMP type I receptor ALK3^QD^. The ALK3^QD ^receptor harbours a Q-to-D point mutation at amino acid 233 in the GS domain thereby replacing the necessary activation by BMPR2-mediated phosphorylation in response to BMP ligand binding [28]. We chose to use OVCA429 cells since they have a BMP receptor expression profile similar to primary OSE and EOC cells (Fig. 1), and exhibit robust BMP4 signalling activity in response to BMP4 treatment (Fig. 2A). We generated five independent clones of 429T-ALK3^QD ^cells, and two of these exhibited low basal expression in untreated cells with readily-detectable ALK3^QD ^expression in response to treatment with 2 μg/mL Dox for 24 h (Fig. 2B). As a functional readout to confirm intact ALK3^QD ^receptor signalling, we observed Dox-induction of *ID1 *and *ID3 *mRNA expression in 429T-ALK3^QD ^cells as compared with 429T controls (Fig. 2C). High basal levels of *ID1 *expression in untreated cells is due to the presence of serum in the growth media required to sustain rtTA expression and Dox-inducible activity in Tet-On cells.

**Figure 3:**
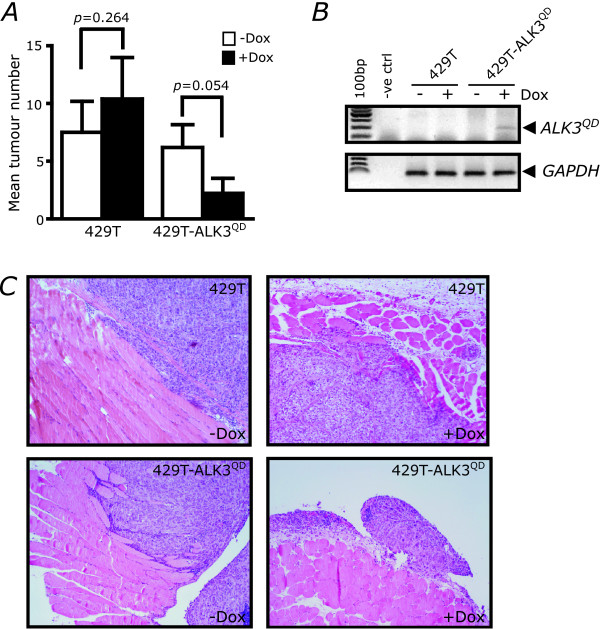
**ALK3^QD ^reduces intraperitoneal tumour formation**. Female CD-1 *nu/nu *athymic nude mice were injected intraperitoneally with a suspension of 5 × 10^5 ^cells (either 429T-ALK3^QD ^or 429T control cells), resulting in four groups of fifteen mice (each cell line with or without Dox-containing chow). (A) Fewer Dox-treated mice injected with 429T-ALK3^QD ^cells developed detectable tumour lesions throughout the peritoneal cavity when compared with Dox-treated 429T-injected mice. (B) *ALK3QD *transgene expression in tumours that formed in nude mice was confirmed by RT-PCR analysis of total RNA with human *GAPDH *mRNA expression serving as a control for xenograft material present in each sample. (C) Tumour specimens isolated from 429T-injected and 429T-ALK3^QD^-injected mice fed a Dox-containing or normal chow diet were analyzed histologically by H&E staining. Tumour implants from the peritoneal wall were adherent to the surface of smooth muscle in Dox-treated 429T-ALK3^QD^-injected mice, whereas localized invasion was evident in specimens from the other groups of mice. 100 × original magnification.

OVCA429 cells consistently form ascites and intraperitoneal tumour lesions when injected directly into the peritoneal cavity of nude mice [24] thereby providing a useful *in vivo *model to study ovarian cancer metastasis. Female CD-1 *nu*/*nu *mice were injected i.p. with 5 × 10^5 ^cells of 429T-ALK3^QD ^or 429T controls. Mice were subsequently divided into two groups (*n *= 15 per group), a group was either fed a normal chow diet or Dox-containing chow to induce ALK3^QD ^expression. After an experimental endpoint of 90-100 d, tumours formed on several surfaces of the peritoneal cavity and measurable ascites developed in mice in all four treatment groups (Table 1). Notably, the proportion of Dox-treated mice injected with 429T-ALK3^QD ^cells that developed measurable ascites was reduced when compared to control mice (28.6% *versus *40-46.7%). In addition, we observed a decrease in the mean number of macroscopic tumour nodules (≥1 mm) that formed within the peritoneal cavity due to ALK3^QD ^expression (Fig 3A). Together, ALK3^QD ^expression was associated with reduced intraperitoneal tumour and ascites formation compared with controls; however, this difference was not statistically significant. We noted that the number of animals developing tumours in the untreated (minus Dox) 429T-ALK3^QD ^group had a lower mean number of tumours (6.2 ± 2.0) than the 429T control groups. This raises the possibility that ALK3^QD ^is expressed at very low levels in the absence of Dox, undetectable by expression analysis, while still producing a modest functional response. Expression of ALK3^QD ^was confirmed in tumours that developed in Dox-treated 429T-ALK3^QD^-injected mice (Fig. 3B). Macroscopic tumours adherent to the peritoneal wall were observed in tumour-bearing mice, and histologic analysis demonstrated foci of penetration into the underlying smooth muscle (Fig. 3C), but this invasion was not evident in tumours isolated from Dox-treated 429T-ALK3^QD ^mice. The majority of tumour nodules were detected in the adipose tissue of the omentum juxtaposed to the pancreas, and the serosa of the spleen, intestine and liver (Table 1). Interestingly, only one Dox-treated 429T-ALK3^QD ^female mouse harboured experimental metastasis to the omentum, the most common site for tumours in all other groups.

**Table 1:** Summary of ovarian cancer cell xenograft studies in nude mice.

	**429T**		**429T-ALK3^QD^**	
		
	**-Dox**	**+Dox**	**-Dox**	**+Dox**
				
*n *^a^	14	15	15	14
No. of mice with ascites (%)	6 (42.9)	7 (46.7)	6 (40.0)	4 (28.6)
Mean ascites volume (± SEM)	1.5 (± 0.9)	1.0 (± 0.6)	0.7 (± 0.4)	1.1 (± 0.5)
No. of mice with tumours (%)	10 (71.4)	9 (60.0)	7 (46.7)	6 (42.9)
Mean tumour number (± SEM)	7.5 (± 2.7)	10.4 (± 3.6)	6.2 (± 2.0)	2.2 (± 1.3)
Tumour sites (number of mice)^b^	Pw (5)	Pw (8)	Pw (3)	Pw (4)
	Spl (4)	Int (1)	Om (6)	Lv (1)
	Spl (2)	Int (1)	Om (8)	Lv (4)
	Spl (3)	Int (2)	Om (6)	Lv (4)
	Spl (2)	Int (0)	Om (1)	Lv (1)

^a ^- Two mice died (not due to tumour growth; cause unknown) prior to the experimental endpoint resulting in 14 mice for the 429T -Dox group and 14 mice for the 429T-ALK3^QD ^+Dox group.

^b ^- Number of macroscopic tumour nodules (≥1 mm diameter) was scored. Observed tumour sites (*i.e*. adherent tumour nodules on tissue/organ surface) were: peritoneal wall (Pw), spleen (Spl), intestine (Int), omentum (Om) and liver (Lv).

### Constitutive ALK3 receptor signalling reduces EOC cell adhesion *in vitro*

**Figure 4:**
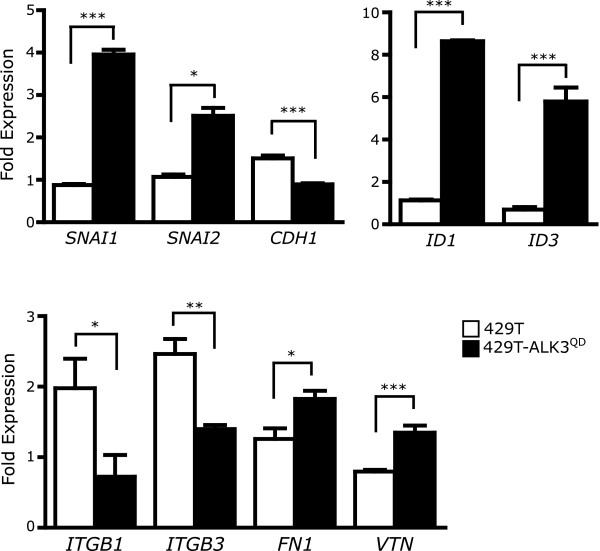
**ALK3^QD ^signalling induces EMT marker expression and reduces β_1_-and β_3_-integrin expression**. Quantitative RT-PCR was performed on total RNA isolated from 429T-ALK3^QD ^cells, and 429T control cells, treated with Dox for 2 d, or left untreated. Human gene-specific primers were used to detect mRNA expression of the EMT markers Snail (*SNAI1*), Slug (*SNAI2*), and E-cadherin (*CDH1*). Quantification of expression of beta- integrins [β_1 _(*ITGB1*), and β_3 _(*ITGB3*)], and the extracellular matrix components, fibronectin (*FN1*) and vitronectin (*VTN*) was also performed. Induction of *ID1 *and *ID3 *mRNA expression by ALK3^QD ^served as a positive control. *GAPDH *mRNA expression was used for normalization, and the fold change in mRNA expression was calculated by the ratio of Dox-treated *versus *untreated cell samples.

We next performed a series of experiments to investigate the underlying basis for reduced *in vivo *metastatic potential of ovarian cancer cells due to constitutively-active BMP signalling. A number of genes involved in cancer metastasis are putative and/or known targets for active BMP signalling in ovarian cancer cells[23]. A well-established and distinguishing molecular signature of epithelia-mesenchymal transition and increased malignancy is the upregulated expression of Snail and Slug transcription factors (encoded by *SNAI1 *and *SNAI2*, respectively) and the subsequent downregulation of their target gene *CDH1 *encoding E-cadherin[29,30]. By performing quantitative RT-PCR we determined that Dox-treatment of 429T-ALK3^QD ^cells led to the 4-fold upregulation of *SNAI1 *(Snail) and 2.5-fold upregulation of *SNAI2 *(Slug) when compared with 429T control cells (Fig. 4). In a reciprocal fashion, ALK3^QD ^signalling reduced *CDH1 *expression by more than 40% *versus *control cells. As a positive control for activated BMP signalling, we detected a substantial increase in both *ID1 *(~8-fold) and *ID3 *(~9-fold) mRNA expression in ALK3^QD^-expressing cells (Fig. 4).

Cell adhesion is likely a key checkpoint regulating the efficient spread of ovarian cancer cells during metastasis. Potential molecular targets of ALK3^QD ^signalling mediating the observed reduction in ovarian cancer cell adhesion are genes encoding integrins and/or extracellular matrix (ECM) components. We performed gene expression analyses for several specific integrins and ECM components known to be expressed by OSE and EOC cells[31,32]. Dox-treated 429T-ALK3^QD ^cells possessed a 64% decrease in *ITGB1 *(β_1_-integrin) and 43% decrease in *ITGB3 *(β_3_-integrin) mRNA expression *versus *429T controls (Fig. 4). In addition, we observed a reciprocal elevated expression of ECM genes *FN1 *(fibronectin) and *VTN *(vitronectin) mRNA in ALK3^QD^-expressing cells by 49% and 69%, respectively.

**Figure 5:**
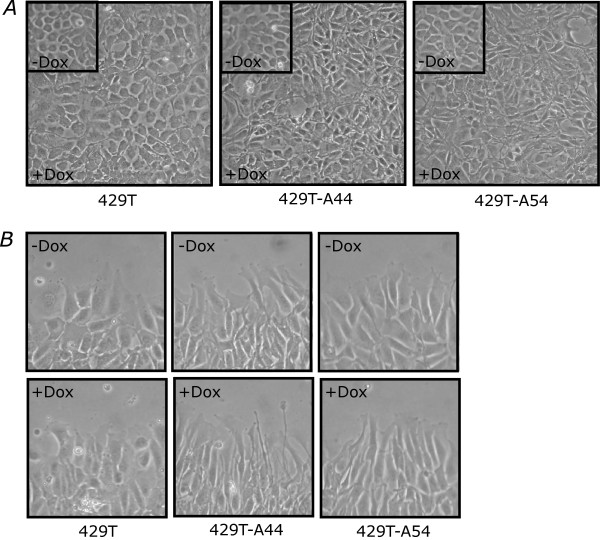
**ALK3^QD ^signalling induces an EMT-like morphology of OVCA429 cells**. (A) Subconfluent monolayer cultures of 429T-A44 and 429T-A54 cells, and 429T control cells, were treated with Dox for 2 d, or left untreated (inset). At confluence, both 429T-A44 and 429T-A54 cells exhibited a greater proportion of refractile, spindle-shaped cells *versus *429T control cells. (B) All cells were grown and treated as described above, but after 2 d a scratch wound was generated. Long cytoplasmic projections extend into the wound space in Dox-treated 429T-A44 and 429T-A54 cells as compared with 429T control cells. Photo images were captured at 100 × original magnification.

Similar to previous observations when primary EOCs and established EOC cell lines were treated with exogenous BMP4 ligand [2,23], induction of ALK3^QD ^did not affect the overall growth rate of OVCA429 cells (data not shown). We observed, however, that when Dox-treated cells reached confluence, both 429T-A44 and 429T-A54 cells had an altered morphology as compared with Dox-treated 429T cells or untreated cells (Fig. 5A). In the untreated state, OVCA429 cells appear as a cobblestone epithelial monolayer. Induction of ALK3^QD ^expression in 429T-A44 and 429T-A54 cells produced a more mesenchymal-like cellular phenotype than uninduced cells, with clusters of refractile, spindle-shaped cells.

To address whether this altered phenotype in response to ALK3^QD ^expression affected the motility of the cells, standard *in vitro *wound-healing assays were performed. There was no difference in motility rates among Dox-treated and untreated 429T, 429T-A44 and 429T-A54 cells (data not shown). When cells were observed during the progress of cell migration (*i.e*. at 24 hours after wounding), Dox-treated 429T-A44 and 429T-A54 cells possessed a more spindle-shaped morphology as they migrated into the manufactured wound space with cells possessing long, cytoplasmic projections extending in the direction of cell movement (Fig. 5B). Interestingly, 429T and 429T-ALK3^QD ^cells are poorly invasive through matrices composed of either rat tail collagen or gelatin, regardless of Dox-treatment (data not shown). This lack of invasiveness is identical to the parental OVCA429 cell line (Mujoomdar & Nachtigal, unpublished observations). Taken together, these data provide further validation that ALK3^QD ^expression in OVCA429 cells results in morphological changes paralleling the observed alterations in EMT marker expression.

**Figure 6:**
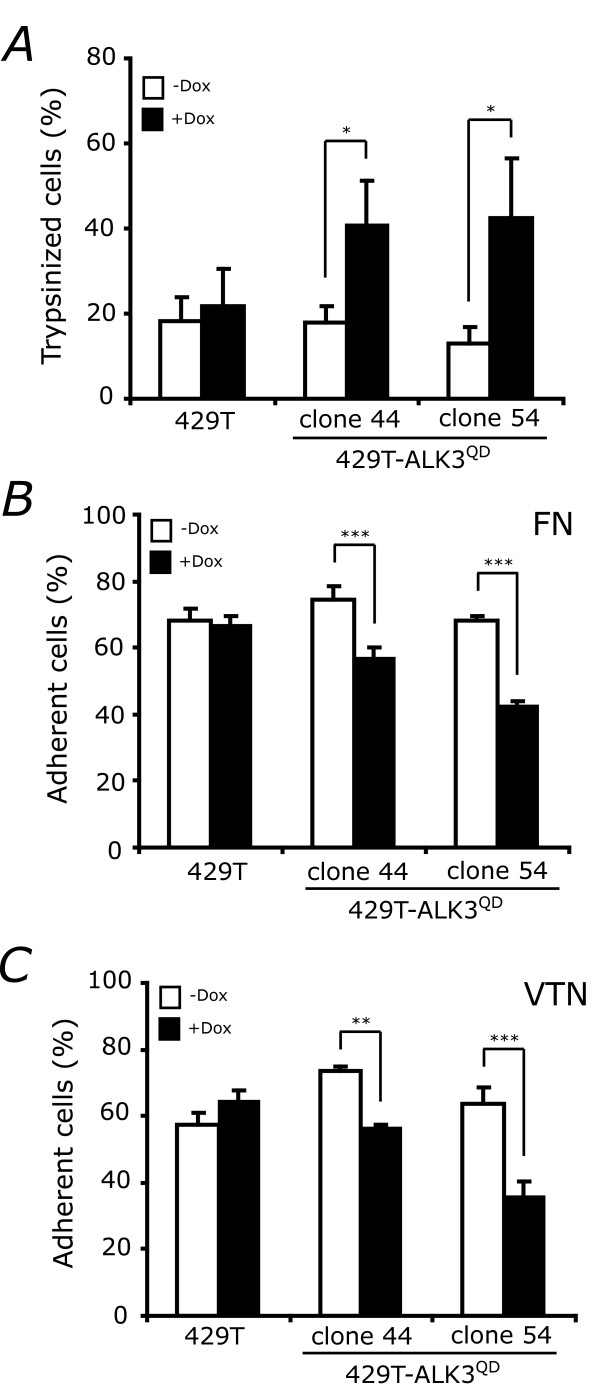
**ALK3^QD ^signalling decreases OVCA429 cell adhesion**. (A) ALK3^QD ^expression increases trypsin-sensitivity of OVCA429 cells. Subconfluent monolayer cultures of 429T-ALK3^QD ^cells (clones A44 and A54), and 429T control cells, were treated with Dox for 2 d (+Dox), or left untreated (-Dox). At confluence, cells were gently trypsinized for 2 min, and the number of suspended cells was scored. The final proportion of released cells was significantly higher in Dox-treated 429T-A44 and -A54 cells *versus *429T control cells. (B,C) ALK3^QD ^expression decreases the adhesion of OVCA429 cells to the ECM components fibronectin (FN) and vitronectin (VTN). Cells were cultured in the presence or absence of Dox, radiolabelled with ^3^H-amino acids, trypsinized, counted, and allowed to recover in serum-containing media. Cells were then seeded at 1 × 10^5 ^cells per well that were pre-coated with FN and VTN and number of attached cells were quantified. Dox-treated 429T-A44 and -A54 cells had a significantly-reduced ability to attach to both FN and VTN as compared with untreated cells, and the 429T control cells. (*, p < 0.05; **, p < 0.01; ***, p < 0.001)

A critical step controlling ovarian cancer metastasis is the ability of EOC cells to adhere to surfaces at secondary sites [1]. The ALK3^QD^-mediated decreases in β_1_- and β_3_-integrin mRNA expression in 429T-ALK3^QD ^ovarian cancer cells would predict a decrease in cell adhesion. Timed cell-detachment assays demonstrated that Dox-treated 429T-ALK3^QD ^cells have increased sensitivity to trypsinization when compared to control cells (Fig. 6A). To directly investigate the adhesive properties of these cells to specific substrates, we performed adhesion assays using tissue culture dishes precoated with the extracellular matrix (ECM) components fibronectin (FN) and vitronectin (VTN), which are produced by EOC cells[32]. As compared to 429T control cells, 429T-ALK3^QD ^cell lines had a reduced ability to attach to FN- and VTN-coated wells when induced to express ALK3^QD ^(Fig. 6B&C).

**Figure 7:**
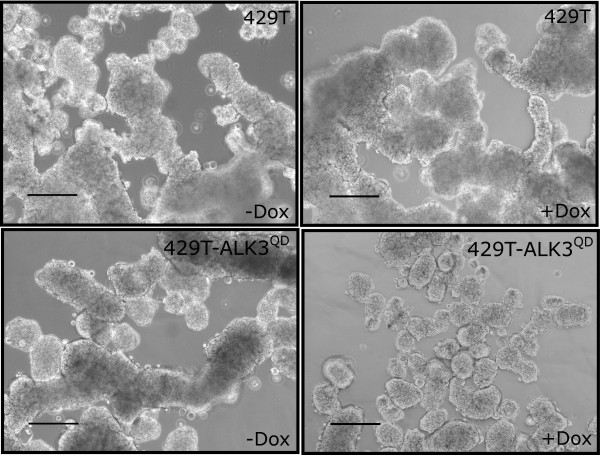
**ALK3^QD ^signalling reduces OVCA429 spheroid formation**. 429T-ALK3^QD ^cells and 429T control cells were grown on Ultra-Low Attachment cluster plates for 2 d while treated with Dox or left untreated. ALK3^QD^-expressing cells produced smaller multicellular aggregates, or spheroids, than untreated 429T-ALK3^QD ^cells and 429T controls. Scale bar = 200 μm.

Multicellular aggregates of EOC cells, termed spheroids, are present in the ascites of patients and are hypothesized to play an essential role in ovarian cancer metastasis [33]. Cell-cell cohesion in spheroids is mediated primarily by cadherins junctions and integrin interactions [34]. Thus, we sought to determine whether the reduced E-cadherin and β1-/β3-integrin expression due to ALK3^QD ^signalling affects the ability of OVCA429 cells to form spheroids. After culturing cells for 2 d on Ultra-Low Attachment cluster plates, Dox-treated 429T-ALK3^QD ^spheroids were smaller in size and less dense-appearing than untreated cells and 429T control cells (Figure 7).

## Discussion

Primary cultures of normal human OSE cells and EOC cells possess an intact BMP4 signalling pathway, yet there are important differences between the response of normal OSE cells to exogenous BMP4 and that of EOC cells [2,23]. For example, primary human EOC cells achieve higher levels of BMP4-induced *ID1 *and *ID3 proto*-oncogene expression than do normal human OSE cells (~10-15 fold in EOC cells, compared to 2-3 fold in normal OSE)[2]. The differential response to BMP4 signalling between OSE and EOC cells is unlikely to be due to altered BMP4 receptor expression levels since we observed no significant differences in the mRNA level of *ALK3 *or *BMPR2*, and expression of *ALK6 *mRNA was largely undetectable in all primary cell samples. Additionally, BMPR2, Smad1 and Smad5 protein levels were similar in primary OSE and EOC cell samples (Shepherd & Nachtigal, unpublished observations).

To observe the effect of autonomous BMP signalling in EOC cells, we chose to express the constitutively-active mutant BMP type I receptor ALK3^QD ^in OVCA429 ovarian cancer cells. Several studies have used mutant BMP receptor expression to obtain insight into the role of BMP signalling in human cancer cells. For example, dominant-negative BMPR2 affects the growth of human breast cancer cells *in vitro *by blocking cells in G_1 _of the cell cycle[35]. Constitutively-active ALK6^QD ^receptor expression decreases the proliferation of human prostate cancer cells as well as their ability to form tumours in nude mice[36], whereas blocking BMP signalling by expression of dominant-negative BMPR2 enhances prostate cancer tumorigenicity[37]. No mutant BMP receptors have been identified in primary human EOC cells, however an intact autocrine BMP4 pathway exists to induce EOC cell spreading, and increased adhesion, motility and invasion[2,23]. Moreover, cell migration is greatly reduced by blocking this autocrine BMP4 signalling pathway with the BMP2/4 antagonist Noggin [23]. Established human EOC cell lines are responsive to exogenous BMP4 ligand at the level of target gene expression, yet appear particularly unaffected in terms of other phenotypic changes observed in primary EOC cell samples from patients[2,23]. By utilizing the inducible expression of a constitutively-active ALK3^QD ^receptor in OVCA429 ovarian cancer cells, we provide additional evidence for the contribution of BMP signalling to affect cellular morphology and adherence in EOC cells, and now extend these findings with direct analysis of its impact on EOC metastasis *in vivo*.

In this report we demonstrate that constitutive BMP signalling through the mutant ALK3 receptor induces EMT markers, consistent with our findings observed in primary EOC cells[23]. EMT is commonly associated with aggressive cancer cell behaviour[38]. Indeed, ectopic overexpression of Snail and Slug in the SkOV3 human ovarian cancer cell line enhances their motility, invasiveness and tumorigenicity[39]. The precise role for EMT in ovarian cancer pathogenesis, however, is not straightforward [40]. Although decreased E-cadherin expression is a hallmark of EMT and is usually correlated with a higher degree of malignancy in most other carcinomas, forced overexpression of E-cadherin in immortalized human OSE cells enhances pre-neoplastic features *in vitro *and establishes tumour-forming ability *in vivo *[41,42]. Our data independently supports this finding since ALK3^QD ^expression led to morphological alterations and changes in gene expression consistent with EMT *in vitro*, yet resulted in a decreased ability to produce ascites and form tumours *in vivo*. Perhaps the functional role of EMT in ovarian cancer is an exception to norm among epithelial-derived malignancies[40] and represents a key process underlying the unique mode of metastasis, *i.e*. direct dissemination into the peritoneal cavity, observed in this type of carcinoma[1,33].

ALK3QD-expressing OVCA429 cells exhibited decreased ability to form spheroids as well as reduced cell-substratum adhesion with concomitant alterations in the expression of a number of genes encoding integrins and ECM components. Le Page and colleagues have recently demonstrated that BMP2 treatment of several ovarian cancer cell lines also reduces the cell-cell cohesion during spheroid formation [43]; however, evidence for the molecular mechanism was not presented. BMP signalling has been shown to alter the expression of integrins and their substrates present in the ECM in several cell types, including EOC cells. For example, α_5_β_1 _and α_v_β_3 _expression is increased in osteoblasts in response to BMP signalling resulting in increased mesenchymal cell adherence[44-46]. Altered expression of β-integrins has numerous implications in human cancer pathogenesis. Conditional loss of β_1_-integrin reduces mammary tumour formation in transgenic mice[47]. Specific to ovarian cancer, it has been postulated that β_1_-integrin expression by EOC cells functions during metastasis to promote cell adhesion to the peritoneal mesothelial surfaces during tumour implantation[48-50]. In addition, blocking β_1_-integrin with interfering antibodies disaggregates EOC spheroids and reduces cell adhesion, spheroid formation, and attachment to peritoneal surfaces [48,51]. Coordinated expression of β_3_-integrin is also involved in EOC cell adhesion, and β_3_-integrin interaction with vitronectin via α_v_-integrin promotes EOC cell proliferation, motility, and ECM degradation[52,53]. In contrast, Kaur and colleagues recently demonstrated that forced α_v_β_3_-integrin overexpression in SkOV3ip1 cells increases cell adhesion *in vitro *yet reduces both invasion and their ability to form secondary tumours in mice; clinical data from this same report implicates that α_v_/β_3_-integrin expression may represent a favourable prognostic marker in ovarian cancer [54]. Ascites-derived primary human EOC cells treated with BMP4 leads to increased β_1_- and β_3_-integrin mRNA expression and correlates with increased adherence to a variety of ECM substrates *in vitro *[23]. In this report, we propose that the downregulation of β1- and β3-integrins caused by an aberrant constitutively-active BMP signalling pathway in a more malignant variant of EOC cells (*i.e*. the OVCA429 cell line) decreases cell adhesion *in vitro *and thereby leads to reduced ascites and intraperitoneal tumour formation *in vivo*. Whether BMP signalling regulates β_1_- and β_3_-integrin expression directly or indirectly to affect EOC cell adhesion during specific steps of ovarian tumorigenesis requires further investigation. The recent work by Kaur et al specifically examined ectopic overexpression of β_3_-integrin in SkOV3ip1 cells[54], whereas other studies have evaluated endogenous integrin expression and function within several other EOC cell lines (CaOV3, SkOV3, OVCAR3, OVCAR5, SW626, OV-MZ-6, 36M2)[48-53]. The majority of these studies suggest that intact integrin function inherent to EOC cells is necessary for adhesion to ECM and peritoneal surfaces in the promotion of EOC metastasis. From our standpoint, it is imperative to perform future studies using both ascites-derived primary EOC cells and established EOC cell lines to help clarify the mechanisms underlying the observed differences in integrin-mediated cell adhesion on the malignant behaviour among these cell types.

Differential effects of BMP signalling *in vitro *compared to *in vivo *have been observed in other tumour models. For example, Langenfeld and colleagues demonstrate that BMP2 induces human lung adenocarcinoma A549 cell proliferation *in vitro *in the presence of serum; but when injected into nude mice BMP2-expressing A549 cells have reduced subcutaneous tumour growth, while development of lung metastases is augmented[55]. They suggest that the cellular response to BMP signalling is dependent upon additional factors in specific tumour microenvironments. Our model has ALK3 signalling constitutively maintained in a cell-autonomous fashion thereby impacting OVCA429 cells directly. From this, we propose that the decreased EOC cell adhesion observed *in vitro *is a critical factor contributing to reduced intraperitoneal ascites and tumour formation *in vivo *as compared with control cells. It will be imperative to further investigate the functional impact of BMP ligands and antagonists on EOC cells *versus *the surrounding tissue microenvironment during ovarian tumour formation and metastasis.

## Conclusions

In summary, we propose that dysregulated BMP signalling is implicated in EOC cell exfoliation from the primary tumour site and subsequent dissemination during ovarian cancer progression. Overactive BMP signalling mediates efficient ovarian cancer cell detachment from the primary tumour; however, at later steps of ovarian cancer progression, down-regulation of BMP signalling may be necessary to establish tumour cell adherence and secondary metastasis formation. To this end, we are currently developing additional *in vivo *models of ovarian cancer to address the implication of modulated BMP signalling on each step from initiation to metastasis.

## Competing interests

The authors declare that they have no competing interests.

## Authors' contributions

TS conceived of the study, generated the inducible cell lines, performed the nude mouse xenografts, quantitative RT-PCR analysis, cell motility, cell adhesion and spheroid formation assays, and drafted the manuscript. MM performed cell motility and invasion assays. MN participated in the study design and coordination and helped to draft the manuscript.

All authors have read and approved the final manuscript.
